# Book and Pamphlet Notices

**Published:** 1859-02

**Authors:** 


### BOOK AND PAMPHLET NOTICES.

A Treatise on Human Physiology; designed for the use of students and practitioners of medicine. By J. C. Dalton, Jr., M.D., Professor of Physiology and Microscopic Anatomy in the College of Physicians and Surgeons, New York; Member of the New York Academy of Medicine; of the New York Pathological Society; of the American Academy of Arts and Sciences, Boston, Mass.; and the Biological Department of the Academy of Natural Sciences, of Philadelphia. With two hundred and fifty-four Illustrations. Philadelphia: Blanchard & Lea. 1859. pp. 608.

Dr. Dalton has given, in the above work, a condensed treatise on human physiology. Though concise, it will be found to present the subject in an inviting and instructive style and form. We like that portion of the work devoted to the elucidation of embryonic development especially well. The illustrations are excellent, and carry the reader through the descriptions of this intricate subject in such an easy manner as to seduce him into a complete reading of it. Students are quite well aware of the exceeding difficulty of getting a knowledge of these processes, as now understood by physiologists, and will be grateful to Dr. Dalton for so greatly facilitating their labors. It is refreshing to look upon the number of new, original and faithful figures of illustration presented us in this volume. Instead of the old, familiar cuts that have descended from Hunter, Munroe, and others of the venerable confraternity of physiological cultivators of the last century, we find two hundred and forty-three new plates, upon which we may *feast* our eyes, and those who have a taste for physiological knowledge, can satiate their appetite for viands of this kind.

Practitioners who have not the time or inclination to wade through the ponderous tomes of Carpenter, can get here a very complete work on physiology, divested of all superfluities, and produced in a very happy and pleasing manner. It may be bought of W. B. Keen, and perhaps others of our bookstores.

The Science and Art of Surgery; being a Treatise on Injuries, Diseases and Operations. By John Erickson, Professor of Surgery and Clinical Surgery in University College, and Surgeon to University College Hospital. An improved American, from the second enlarged and carefully revised London edition. Illustrated by four hundred and seventeen engravings on wood. Philadelphia: Blanchard & Lea. 1859. With about one thousand pages.

This work is written with the spirit and originality characteristic of its author, and has already become a favorite with American surgeons. Its excellencies are so plainly shown in this edition, that they will insure a continuance of its popularity. In looking over its pages for the names and accredited labors of American surgeons, we are struck with the difficulty with which knowledge crosses the Atlantic, against the east winds originated on the European side of the waters. We hope it will not be long that it may be justly considered a misfortune to a scientific man, that he was born in America. The tide of intelligence and science has so nearly encircled the globe, that we think it will not be a great while before the sun of science will rise to the world from our continent; then our medical men will be considered as near the fountain of *all* learning.

A Practical Treatise on the Diseases of Children. By D. Francis Condie, M.D., Fellow of the College of Physicians, Member of the American Medical Association, Member of the American Philosophical Society, etc. Fifth edition, enlarged and revised. Philadelphia: Blanchard & Lea. pp. 762. For sale by W. B. Keen.

We need not speak of the merits of this work. It is, as it deserves to be, in the library of almost every intelligent physician in the West, and our readers are too well aware of its many excellencies to require enlightenment on this matter. It will only be necessary to say to them, that this edition is so much improved, that they will be under the necessity of having it, if they wish to keep up with the times. It has its own peculiar merits, which cannot be supplied or supplanted by other works of the kind.

The Functions of the Cerebellum.—A pamphlet, of nineteen pages, by Dr. E. Andrews, upon this subject, has been laid upon our table. It first appeared among the transactions of the "American Medical Association," at the session in 1858. It is well written, without ornament or display, but a compilation of facts, which fell under the author's observation, from his own dissections. His theory is, That the median lobe presides over the motor power of the muscles of the inferior extremities; to support which he presents two propositions :

1. In the warm-blooded animals, the median lobe, or vermiform process of the cerebellum, varies in size directly as the bulk and power of the anterior group of muscles.

2. The lateral lobes vary in like manner, as the power of the posterior group of muscles; subject, however, to certain variations hereafter to be mentioned.

As illustrations of his theory, and reasons for his conclusions, he examples the cerebellum of man; the common gull; dolphin, and other animals; demonstrating clearly the connections between his facts and theory. Physiology long since pointed out the cerebellum as the centre of muscular innervation, and experiments prove that the organ may be seriously injured without impairing any vital function except motion. The author of the pamphlet before us does not enter into experimental or pathological evidences, intimating his intention to make those proofs the subject of another report, to be issued hereafter. In the present work he restricted himself to the evidences derived from comparative anatomy. One point of great interest which he brings up, is, that the commonly-received opinion that the cerebellum is simply a *co-ordination* of motion, is utterly without foundation in comparative anatomy. His figures show that the size of the cerebellum has no relation whatever to the number and variety of co-ordinated actions of the animal, but, on the contrary, is directly in proportion to the bulk and power of muscle, and to the amount of intelligence which regulates it, in total disregard of the variety of combinations. In support of this position, the author institutes comparisons between the cat and the seal, the mouse and the bird, etc., showing numerous instances where the animal possessing the smallest cerebellum exercises the greatest co-ordinating capacity.

Dr. A. traces, in the lateral lobes of the cerebellum, a double relation. Its size varies, first, as the muscular power of the posterior extremeties; and secondly, as the development of the hemispheres of the cerebrum; or, in other words, as the grade of intelligence of the animal. This view is illustrated by several engravings.

The phrenological doctrine, that the cerebellum is the seat of the generative instinct, is passed over without any notice.

